# No rest for elderly femur fracture patients: early surgery and early ambulation decrease mortality

**DOI:** 10.1186/s10195-020-00550-y

**Published:** 2020-08-30

**Authors:** Alessandro Aprato, Marco Bechis, Marco Buzzone, Alessandro Bistolfi, Walter Daghino, Alessandro Massè

**Affiliations:** School of Medicine, University of Turin, Viale 25 Aprile 137 Int 6, 10133 Turin, Italy

**Keywords:** Femur fracture, Early treatment, Walking

## Abstract

**Background:**

Literature has shown a significant correlation between early treatment and mortality in femur fractures, but the influence of time to ambulation on mortality has not been studied. The purpose of the present study is to evaluate whether time to ambulation is correlated to femur fracture mortality independently from time to surgery.

**Patients and methods:**

All patients older than 65 years admitted at a level I trauma center with proximal femoral fracture during a 1-year period were included. The following data were collected: age, gender, date and time of admission to emergency department, height, weight, body mass index, type and side of fracture, ASA score, date and time of surgery, surgical time, time to ambulation, length of hospitalization, death during hospitalization, and mortality at 6 and 12 months.

**Results:**

The study sample comprises 516 patients. The mean age was 83.6 years; ASA score was 3–5 in 53% of patients; 42.7% presented with medial fracture; mean time between admission and surgery was 48.4 h; 22.7% of patients were not able to walk during the first 10 days after fracture; mean duration of hospitalization was 13 days; and mortality was 17% at 6 months and 25% at 1 year. Early surgery and walking ability at 10 days after trauma were independently and significantly associated with mortality at 6 months (*p* = 0.014 and 0.002, respectively) and at 1 year (0.027 and 0.009, respectively).

**Conclusions:**

Early surgery in femur fracture became a priority in health systems, but early postoperative physiotherapy also plays a major role in prevention of mortality: independently from surgical timing, patients who did not walk again within 10 days from surgery showed mortality rates higher than those of patients who did.

**Level of evidence:**

IV.

## Highlights

1. Patients who do not walk again within 10 days from surgery have mortality rates almost double than those of patients who do (6% versus 11% at 6 months and 7% versus 17% at 1 year).
2. Walking ability at 10 days after trauma is significantly associated with mortality at 6 months and at 1 year independently from early surgery (within 48 h).

## Introduction

Hip fractures in the elderly represent a major public health burden worldwide [1], and aging of population is increasing its rate [2, 3]. Mortality is reported to be up to 25% at 1 year [4], and several changeable factors may contribute to this event [5]. Literature has shown a significant correlation between early treatment and mortality, but so far, the influence of time to ambulation on mortality has not been studied, although immobilization in bed is a well-known risk factor for complications. The aim of this study is to evaluate whether time to ambulation is correlated to femur fracture mortality independently from time to surgery.

## Patients and methods

### Settings

All patients admitted at a level I trauma center with proximal femoral fracture during a 1-year period were included in this study. Exclusion criteria included patient being younger than 65 years, death before surgery, nonsurgical treatment, and inability to walk before trauma.

### Data collection

Hospital charts were retrospectively reviewed following patients’ informed consent to use their data. The present study was approved by the institutional review board and was conducted in accordance with the ethical standards laid down in the 1964 Declaration of Helsinki and its later amendments. The following data were collected: age at admission, gender, date and time of admission to the emergency department (ER), height, weight, body mass index (BMI) [6], type of fracture (pertrochanteric, subtrochanteric, basicervical, subcapital, and transcervical; then grouped into intracapsular or extracapsular), side of fracture (right or left), American Society of Anesthesiologists (ASA) score [7], date and time of surgery, surgical time, length of hospitalization, death during hospitalization, ability to walk 10 days after fracture, and mortality at 6 and 12 months. All patients were allowed to full weight bearing after surgery. Ambulation from bed to bathroom with walking aids was the minimum criterion to define the patient as able to walk.

Patients were also divided into two groups according to early (within 48 h) or delayed (> 48 h) surgical treatment to evaluate whether early surgery (among other factors) was significantly related to mortality. All data were analyzed with standard descriptive statistics.

The relationship between mortality and study characteristics was assessed with multivariate regression models. Age and ASA score were considered as continuous variables for the multivariate regression models. *p*-Values lower than 0.05 were considered statistically significant. All analyses were performed using Stata version 12 (Stata Corporation, College Station, TX).

## Results

Out of 583 patients, 67 were excluded (46 were not able to walk before trauma, 11 were younger than 65 years old, 3 underwent a nonsurgical treatment, 2 died before surgery, and 5 presented incomplete data). Therefore, the study sample included 516 patients.

In Table 1, baseline data are presented, while in Table 2, surgical timing, ability to walk before 10 days from trauma, length of hospitalization, and mortality are presented. Comparison between patients who walked within 10 days from injury and patients who did not is presented in Table 3. Among patients who walked again within 10 days from surgery, mortality rates were 6% and 7% at 6 months and 1 year, respectively. Among patients who did not walked again before 10 days from surgery, mortality rates were 11% and 17% at 6 months and 1 year respectively.

**Table 1:** Baseline data

Mean age (years), mean (IQR)	83.6 (75.3–89.9)
Female (%)	74.2
Male (%)	25.8
BMI (kg/m^2^), mean (IQR)	23.7 (19.5–27.9)
Fracture type (%)	
Subcapital	8.8
Basicervical	16.7
Mediocervical	20
Lateral	54.5
ASA 1–2 (%)	47.4
ASA 3–5 (%)	52.6

**Table 2:** Surgical timing, ability to walk before 10 days, and mortality at 6 months and 1 year

Mean time between admission and surgery	48.4 h (IQR 13–78 h)
Patients treated within 48 h from admission	53.0%
Patients able to walk before 10 days	77.3%
Mean duration of hospitalization	13 days (IQR 6–20 days)
Mortality at 6 months	17.0%
Mortality at 1 year	24.7%

**Table 3:** Comparison between patients who walked within 10 days from surgery and patients who walked after 10 days

	Early-walker group	Other	*p*-Value
Mean age (years), mean (IQR)	85.2 (77.8–88.2)	84.6 (74.8–90.2)	0.849
Female (%)	72.4	78.4	0.19
Male (%)	27.6	21.6	
BMI (kg/m^2^), mean (IQR)	23.5 (20.1–28.2)	24.6 (18.5–28.9)	0.385
Lateral fracture (%)	56.0	62.1	0.243
ASA 1–2 (%)	48.8	41.7	0.236
ASA 3–5 (%)	51.2	58.3	

Among patients who walked within 10 days from surgery, 54% were operated within 48 h from admission, while 46% were operated after 48 h. Among patients who did not walked again before 10 days from surgery, 41% were operated within 48 h from admission, while 59% were operated after 48 h. Multivariable analyses are presented in Tables 4 and 5.

**Table 4:** Logistic regression between mortality at 6 months and age, ASA, early surgery, ability to walk 10 days after surgery, gender, and fracture classification

6-Month mortality	Coeff.	Std. err.	*p*-Value	95% confidence interval	
Age	−0.052	0.041	0.208	−0.133	0.029
ASA	0.425	0.255	0.026	0.075	0.926
Surgery within 48 h	0.524	0.332	0.014	0.126	1.174
Ability to walk 10 days after surgery	1.078	0.352	0.002	0.388	1.769
Male	−0.089	0.384	0.817	−0.840	0.663
Intracapsular fracture	0.608	0.336	0.070	−0.050	1.267

**Table 5:** Logistic regression between mortality at 1 year and age, ASA, early surgery, ability to walk 10 days after surgery, gender, and fracture classification

1-Year mortality	Coeff.	Std. err.	*p*-Value	95% confidence interval	
Age	−0.049	0.037	0.186	−0.121	0.024
ASA	0.756	0.231	0.001	0.304	1.209
Surgery within 48 h	0.383	0.291	0.027	0.186	0.952
Ability to walk 10 days after surgery	0.870	0.331	0.009	0.220	1.519
Male	0.458	0.319	0.151	−0.167	1.083
Intracapsular fracture	0.396	0.290	0.171	−0.171	0.964

## Discussion

The main purpose of this study is to evaluate whether time to ambulation is correlated to femur fracture mortality independently from time to surgery.

In our study, the mean time between hospitalization and surgery was 48 h, the mean time from surgery to first walking day was 5 days, with an average of 5.2 days of immobility. Of all patients, 77% were able to walk before 10 days. Global mortality at 6 months was 17%, while at 1 year, this was 25%. These findings are similar to the results of a metaanalysis of systemic reviews [8] that showed a 17% mortality at 1 year in patients with early surgery (< 48 h) versus 21% in patient who waited more than 48 h [9].

Our data showed that patients who did not walk again within 10 days from surgery had mortality rates almost double than patients who did (6% versus 11% at 6 months and 7% versus 17% at 1 year). Furthermore, walking ability at 10 days after trauma was significantly associated with mortality at 6 months and at 1 year independently from early surgery (within 48 h).

Literature have strongly demonstrated a significant correlation between early surgical treatment and mortality. However, recent studies have showed that early surgical intervention without prompt mobilization and weight bearing did not seem sufficient to reduce the hospitalization and recovery time [10, 11]. Our study suggests that, without early ambulation, mortality rates increase in femur fracture patients.

As shown in a previous study [10], our results suggest that the probability of walking again after the surgery is not only influenced by surgical timing but also by ASA score. In fact, comorbidities [5] may influence mortality after a femoral fracture. Furthermore, baseline prefracture locomotion, abnormal clinical findings on admission, dementia, and baseline living situations [5] influence the outcomes. In addition to those, intrahospital factors, rehabilitation schedules, holidays and weekends, together with limited capacity of operating theaters and personnel [12] play a central role delaying the time from admission to surgery and to physiotherapy.

Modifiable factors for an early ambulation can be an optimal pain management (i.e., reducing narcotics and choosing locoregional versus general anesthesia) [13], early removal of urinary catheter, and optimal management of blood transfusions [14].

The study was limited by its retrospective design; furthermore, only patients from a single hospital from a single European country were included; thus, findings may not be generalizable to other geographical regions. Another limit is the choice of timing cutoff: 48 h for early treatment was based on literature data, while 10 days since trauma was arbitrarily chosen.

Mobilization of the patient within 24 h is recommended by numerous international guidelines (GEIOS, NICE, SIGN, SEGG-SECOT, and NZGG) since it is associated with a decrease in comorbidities [15]. Our results suggest that also early postoperative physiotherapy plays a role in prevention of mortality. Therefore, early surgery and early mobilization should be performed to decrease the mortality rate at 6 and 12 months after femur fracture.

Our data showed mortality rates almost double in patients who did not ambulate within 10 day from surgery (6% versus 11% at 6 months and 7% versus 17% at 1 year). Furthermore, ambulation within 10 days after the trauma was significantly associated with increased risk of 6 and 12 months mortality independently from time to surgery (< 48 h). Association of early surgery and early mobilization should be performed to decrease the mortality rate at 6 and 12 months.

## Data Availability

Paper copy of the database is available at Città della salute e della Scienza di Torino
